# Novel Blood Indicators of Progression and Prognosis in Renal Cell Carcinoma: Red Cell Distribution Width-to-Lymphocyte Ratio and Albumin-to-Fibrinogen Ratio

**DOI:** 10.1155/2020/2895150

**Published:** 2020-11-25

**Authors:** Chenjun Ma, Quan Liu, Chengyang Li, Jiwen Cheng, Deyun Liu, Zhanbin Yang, Haibiao Yan, Bo Wu, Yongxian Wu, Jiawen Zhao

**Affiliations:** ^1^Department of Urology, The First Affiliated Hospital of Guangxi Medical University, Nanning, Guangxi, China; ^2^Department of Urology, Liuzhou Traditional Chinese Medical Hospital, Liuzhou, Guangxi, China

## Abstract

**Objective:**

To evaluate the value of preoperative red cell distribution width-to-lymphocyte ratio (RLR) and albumin-to-fibrinogen ratio (AFR) to the prognosis of patients after renal cell carcinoma (RCC).

**Methods:**

From 2012 to 2016, a total of 273 RCC patients underwent radical nephrectomy or partial nephrectomy. This study retrospectively analyzed this group of patients. X-tile software was used to determine the optimal values of RLR and AFR in the peripheral blood. The nomogram constructed with independent factors was used to predict the survival outcome of the patients after RCC.

**Results:**

The RLR of the RCC group was higher than that of the normal control group (*P*=0.002), whereas the AFR of the RCC group was lower than that of the normal control group (*P* < 0.001). RLR and AFR are related to tumour type and tumour-node-metastasis (TNM) stage (*P* < 0.05 for all). Cox regression analysis showed that the independent prognostic factors affecting overall survival and disease-free survival in the RCC group were symptom, tumour type, TNM stage, Fuhrman grade, RLR, and AFR (*P* < 0.05 for all). The nomogram constructed by multiple factors has better predictive power for patients after RCC.

**Conclusion:**

Preoperative RLR and AFR can serve as potential biomarkers to predict the prognosis of postoperative RCC patients and improve the predictability of patient recurrence and survival.

## 1. Introduction

Primary kidney neoplasm is one of the top 10 most common malignant tumours in the United States, with 73,750 new cases estimated and 14,830 deaths estimated according to Global Cancer Statistics 2020 by the American Cancer Society [1]. Renal cell carcinoma (RCC) stems from the renal cortex and is the most common primary kidney neoplasm. When RCC is diagnosed, about 30% of RCC patients are already in the advanced stage of the disease; therefore, the risk of postoperative recurrence is high [2]. Even in patients with local RCC, about one-third of patients after surgery may have cancer metastasis [3]. Despite advances in treatment, the prognosis of patients with advanced RCC is still not satisfactory. The current prognostic assessment system is still not perfect and has been primarily verified among Caucasian ethnicities, such as the tumor-node-metastasis (TNM) stage; UCLA-integrated staging system; and Mayo Clinic stage, size, grade, and necrosis (SSIGN). Therefore, the methods of prognostic assessment must be continuously improved [4, 5].

Numerous clinical and experimental studies have convincingly supported the concept that inflammation is an important component of tumour progression. Red blood cell distribution width (RDW) reflects the size variability of red blood cells and is routinely measured for anaemia or blood disease. The prognostic role of RDW in several solid tumours has been observed [6, 7]. Elevated levels of RDW may reflect systemic inflammation supporting tumour progression. Peripheral blood and tumour-infiltrating lymphocytes are important effector mechanisms of antitumour immunity. We have conducted a systematic review and meta-analysis that low pretreatment lymphocyte count may represent an unfavourable prognostic factor for clinical outcomes in patients with solid tumours [8]. RDW-to-lymphocyte ratio (RLR), a combination of the two parameters, is easily acquired using blood routine tests. To the best of our knowledge, this study is the first to analyse the prognostic role of RLR in patients with RCC. Serum albumin has protective effects such as nutrition and anti-inflammatory, and fibrinogen can promote tumor cell invasion and metastasis through epithelial-mesenchymal transition and induce tumor blood vessel formation, thereby participating in tumor progression [9, 10]. Elevated serum concentrations of fibrinogen and decreased serum concentrations of albumin are markers of elevated systemic inflammation, and elevated FAR might be associated with a worse prognosis [10–12]. We retrospectively investigated peripheral blood cell counts in south Chinese patient cohorts to explore the relative biological contributions of RLR and albumin and fibrinogen (AFR), two novel indicators in patients with RCC, in a comprehensive fashion across all RCC stages and pathologic subtypes. Their results are readily available because plasma fibrinogen, albumin, red blood cell distribution width, and lymphocytes are routinely used as preoperative markers. We further searched for factors related to the development and progression of RCC. Based on RLR, AFR, and clinical-pathologic data, we establish a clearly preferable nomogram to predict the survival of patients with RCC.

## 2. Materials and Methods

### 2.1. Clinical Data

A retrospective analysis was performed on 273 patients who underwent nephrectomy or partial nephrectomy in the First Affiliated Hospital of Guangxi Medical University from 2012 to 2016 and were pathologically confirmed as RCC after surgery. The exclusion criteria were as follows: (1) abnormal liver function; (2) bilateral RCC; (3) acute or chronic inflammation; (4) autoimmune diseases; (5) hematologic diseases; (6) other malignancies; and (7) no complete clinical and pathologic data. Patient information includes general information (gender, age, and BMI), haemoglobin (HGB), serum calcium (Ca), creatinine, endogenous creatinine clearance (Ccr), basic disease (hypertension, diabetes, coronary heart disease, and thyroid dysfunction), symptom (haematuria, low back pain, and abdomen mass), surgery (open or laparoscopic surgery), tumour site, tumour size (the maximum diameter of the tumour), tumour necrosis, tumour type, tumour stage, Fuhrman grade, preoperative blood test result (absolute lymphocyte count, RDW, serum albumin, and fibrinogen). In our study, RDW refers to RDW-CV. Tumours were staged by a staging system issued by the American Joint Committee on Cancer in 2010 [13] and graded by Fuhrman classification [14]. Tumour necrosis is defined as the presence or absence of coagulation-type necrosis in the tumor under the microscope. RLR is defined as RDW/absolute lymphocyte count∗100. AFR is defined as serum albumin/fibrinogen. Overall survival (OS) is defined as the postoperative death to any cause or the end of follow-up, and disease-free survival (DFS) is defined as the date of first relapse (locally or remotely) or the time of death after surgery.

All patients were followed up for routine physical examination, laboratory examination, and necessary imaging. Review was performed once every half year for the first 2 years and once every year after 2 years [15]. All patients signed written informed consent, and the study was approved by the hospital ethics committee.

### 2.2. Detection of Peripheral Blood Cell Count, RDW, Serum Albumin, and Fibrinogen

Peripheral blood of all patients was collected with ethylenediaminetetraacetic acid tube a week before surgery. All peripheral blood samples were collected from 7 : 30 am to 9 : 30 am in the morning. A Beckman Coulter LH-780 whole blood cell analyser was used to detect lymphocyte count and RDW, a Hitachi 7600 was used to detect serum Alb, and an ACL TOP haemagglutinometre was used to detect Fib.

### 2.3. Statistical Analysis

**Figure 1:**
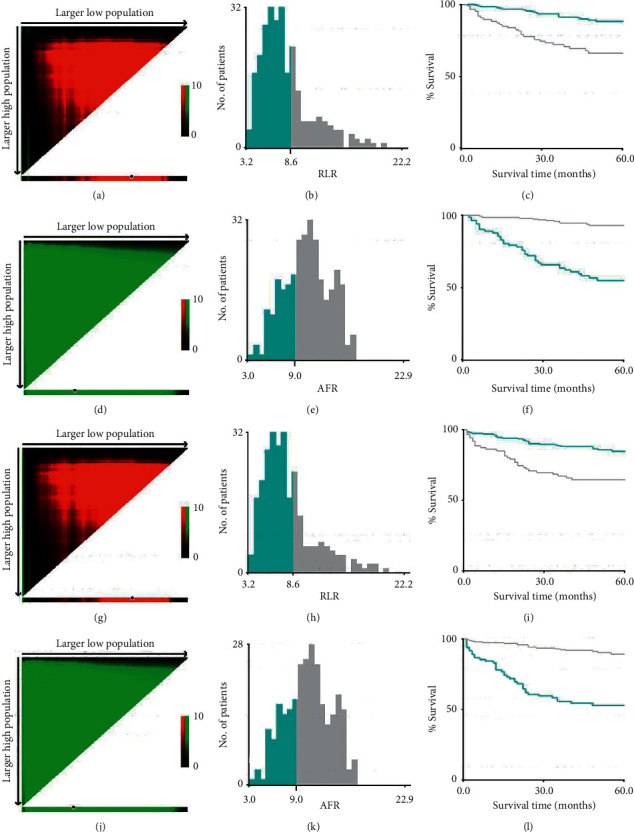
The 5-year OS (AF) and DFS (G-L) were *x*-tile analyzed using patient data to determine the optimal cutoff value for blood RLR and AFR. (a, d, g, j) The data are represented by the panel graph in different colors to indicate possible cutoff values. The best cutting point (8.8 and 9.0, respectively) is determined by the black circle on the *x*-tile image and shown in the histogram in the middle. (b, e, h, k) The histograms of the distribution of the number of people in RLR and AFR, and the kaplanMeier curves of OS (c, f) and DFS (i, l) show the difference in survival of different groups of RLR and AFR.

The clinical data of RCC patients were statistically analyzed using X-tile, SPSS 22.0, Graphpad-Prism 6.0 and R 3.6.3 software. X-tile was used to determine the optimal cut-off of RLR and AFR in patients with RCC (Figure 1). Graphpad-Prism software was used to compare the data of healthy people and patients with RCC. The clinical data of patients were compared by *T* test, chi-squared test, or rank sum test. The Kaplan–Meier method was used to calculate the OS and DFS rates. The log-rank test was used to compare the significance of OS and DFS rates between groups. Univariable and multivariable analyses were performed using the Cox proportional hazards model. With *P* < 0.05 as a reference, Cox regression analysis was used to screen the single factors that are important to the prognosis of RCC patients. The “rms”, “Hmisc,” and “survival” data packages of R 3.6.3 software were used to create the nomogram. The model could verify its accuracy through a *C* index and calibration plot. All statistics were two-sided, and statistical significance was considered at *P* < 0.05.

## 3. Results

### 3.1. General Clinical Characteristics

**Figure 2:**
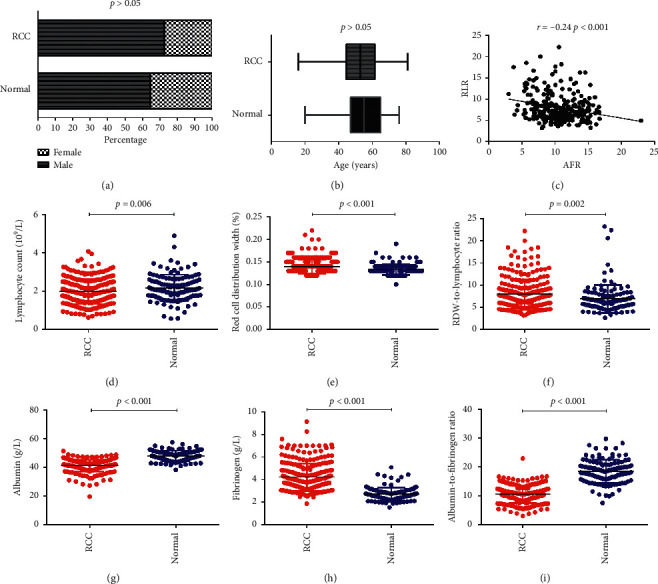
Blood cell counts from healthy people and patients with RCC. (a, b) There was no significant difference in age and gender between NVs and patients with RCC (both *P* > 0.05). (c) Correlations of RLR with AFR in RCC patients. The lymphocyte counts (d), albumin (g), and albumin-to-fibrinogen ratio (i) in patients with RCC were significantly lower than those in healthy people. The red cell distribution width (e), RDW-to-lymphocyte ratio (f), and fibrinogen (h) in RCC patients were significantly higher than those in healthy people.

The median follow-up time for this study was 50.5 months. Among the 273 patients, the 1-year, 3-year, and 5-year OS and DFS rates were 95.2%, 85.1%, and 81.2% and 91.5%, 81.4, and 78.2%, respectively. The clinical characteristics of 273 patients with RCC are presented in Table 1. At the same time, we selected 120 healthy people as controls, including 77 males and 43 females. No significant difference in age (*P* > 0.05, Figure 2(a)) and gender (*P* > 0.05, Figure 2(b)) was found between the patients with RCC and the healthy medical examiners, and the lymphocyte count of the patients with RCC was lower than that of the healthy subjects (*P*=0.006, Figure 2(d)). The RDW (*P* < 0.001, Figure 2(e)) and RLR (*P*=0.002, Figure 2(f)) of the patients with RCC were significantly higher than those of the healthy subjects. Compared with the healthy subjects, the patients with RCC had lower albumin and AFR (*P* < 0.001, Figures 2(g) and 2(i)) and higher fibrinogen (*P* < 0.001, Figure 2(h)). At the same time, AFR and PLR showed a negative correlation (*r* = −0.24, *P* < 0.001, Figure 2(c)). The comparison of clinical and pathologic data between the two groups of patients in PLR and AFR is presented in Table 2. The RLR level is related to age, BMI, HGB, Ccr, basic disease, symptom, tumour size, tumour type, and TNM stage. The AFR level is related to age, gender, HGB, creatinine, Ccr, symptom, surgery, tumour size, tumour type, TNM stage, and Fuhrman grade.

### 3.2. Univariable Analysis

With *P* < 0.05 as a reference, Cox regression analysis was used to screen the single factors that are important to the prognosis of RCC patients. Potential risk factors for OS by the univariable Cox analysis including age (*P*=0.026), HGB (*P*=0.001), serum Ca (*P*=0.019), Ccr (*P* < 0.001), symptom (*P*=0.001), surgery (*P*=0.001), tumour size (*P* < 0.001), tumour type (*P* < 0.001), TNM stage (*P* < 0.001), Fuhrman grade (*P* < 0.001), RLR (*P* < 0.001), and AFR (*P* < 0.001) were enrolled into the multivariable Cox analysis (Table 3). Potential risk factors for DFS by the univariable Cox analysis including HGB (*P*=0.004), Ccr (*P*=0.001), symptom (*P*=0.001), surgery (*P*=0.002), tumour size (*P* < 0.001), tumour type (*P* < 0.001), TNM stage (*P* < 0.001), Fuhrman grade (*P* < 0.001), RLR (*P* < 0.001), and AFR (*P* < 0.001) were enrolled into the multivariate Cox analysis (Table 3).

**Figure 3:**
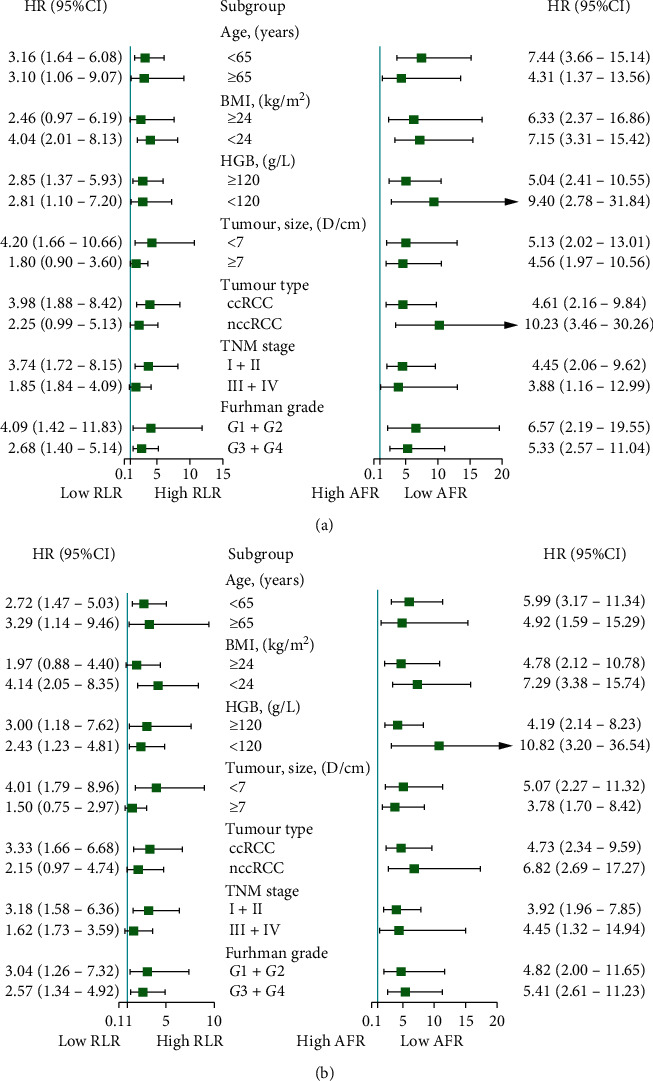
Forest plot showing OS (a) and DFS (b) according to subgroup effects. HR, hazard ratio; CI, confidence interval.

Figure 3 shows the results of the hierarchical analysis. We performed stratified analysis on age, BMI, HGB, tumour size, tumour type, TNM stage, Fuhrman grade, and so on. The results indicated that in the subgroups of BMI ≥ 24, tumour size ≥27, nccRCC, and stages III–IV, the survival time of the patients with high RLR showed no difference from that of the patients with low RLR, whereas the survival time of the patients with high RLR in the other subgroups was shorter than that of the patients with low RLR. In all subgroups, the survival time of the patients with low AFR was shorter than that of the patients with high AFR.

### 3.3. Multivariable Analysis

Cox regression analysis showed that the independent prognostic factors affecting OS and DFS in the patients with RCC were symptom (*P*=0.036 for OS, *P*=0.020 for DFS), tumour type (*P* < 0.001 for OS, *P* < 0.001 for DFS), TNM stage (*P* < 0.001 for OS, *P* < 0.001 for DFS), Fuhrman grade (*P* < 0.001 for OS, *P*=0.003 for DFS), RLR (*P*=0.007 for OS, *P*=0.013 for DFS), and AFR (*P*=0.019 for OS, *P*=0.008 for DFS) (Table 4).

**Figure 4:**
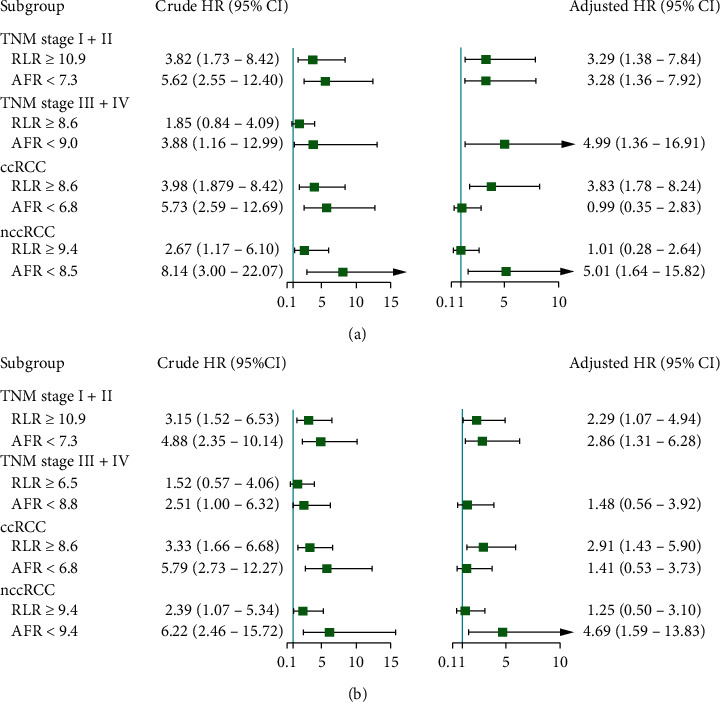
Cox regression forest plot of circulating inflammatory biomarkers showing OS (a) and DFS (b) in each subgroup. HR, hazard ratio; CI, confidence interval.

To further analyse the evaluation value of RLR and AFR in the patients with RCC, we performed a subgroup analysis of tumour types and TNM stages (Figure 4). Results showed that TNM stage I–II (adjusted HR = 3.29, 95% CI = 1.38 – 7.84 for OS, adjusted HR = 2.29, 95% CI = 1.07 – 4.94 for DFS) and ccRCC (adjusted HR = 3.83, 95% CI = 1.78 – 8.24 for OS, adjusted HR = 2.91, 95% CI = 1.43 – 5.90 for DFS) are the independent risk factors affecting the OS and DFS of patients with high RLR, whereas TNM stages I–II (adjusted HR = 3.38, 95% CI = 1.36 – 7.92 for OS, adjusted HR = 2.86, 95% CI = 1.31 – 6.28 for DFS), TNM stages III–IV (adjusted HR = 4.99, 95% CI = 1.36 – 16.91 for OS), and nccRCC (adjusted HR = 5.01, 95% CI = 1.64 – 15.82 for OS, adjusted HR = 4.69, 95% CI = 1.59 – 13.83 for DFS) are the independent risk factors affecting the OS and DFS of patients with low AFR.

### 3.4. Nomogram Plot and C Index

**Figure 5:**
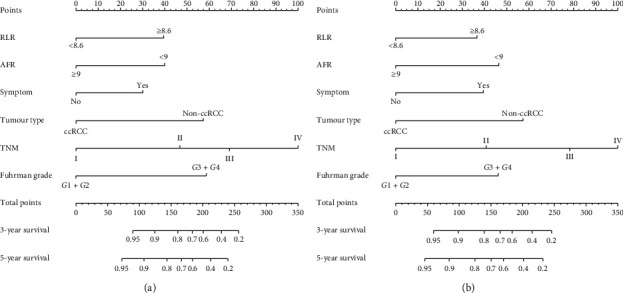
Nomogram to estimate the probability of OS (a) and DFS (b) at 3 and 5 years.

**Figure 6:**
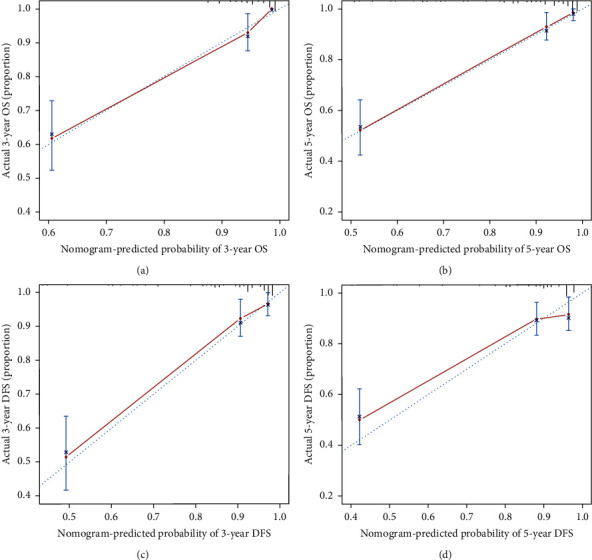
Calibration curves of the nomogram for 3-year OS (a), 5-year OS, (b) 3-year DFS, and (c) and 5-year DFS (d).

**Figure 7:**
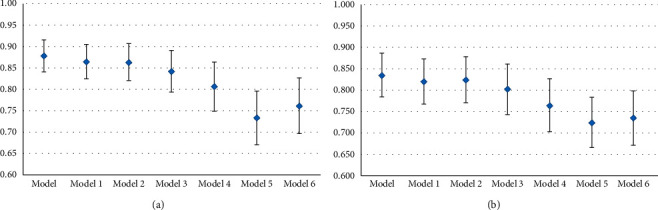
*C* index forest plots of different models. *C* index for predicting the survival probability of (a) OS in 5 years and (b) DFS in 5 years. Model: RLR + AFR + symptom + tumour type + TNM + Fuhrman grade; Model 1: RLR + symptom + tumour type + TNM + Fuhrman grade; Model 2: AFR + symptom + tumour type + TNM + Fuhrman grade; Model 3: symptom + tumour type + TNM + Fuhrman grade; Model 4: TNM + Fuhrman grade; Model 5: symptom + tumor size; Model 6: TNM.

In order to more accurately and effectively predict the survival rate of RCC patients, we used the independent prognostic factors of OS and DFS in the patients with RCC to establish a nomogram plot based on the COX regression model analysis (Figure 5). The model we constructed could more accurately predict the prognosis of patients, and the higher the weighted score of all factors in the model, the worse the prognosis of patients. The calibration curve of the nomogram was close to the reference line, indicating that the model performs well under internal verification (Figure 6). The *C* index is used to evaluate the accuracy of the predictive nomogram, and the higher the index, the more accurately the model can assess the clinical outcome of patients with RCC. Compared with the *C* index of other models for predicting the *C* index of RCC patients with 5 year OS and 5 year DFS, our nomogram had the highest *C* index and the accuracy of prediction was more advantageous. In addition, RLR and AFR were conducive to nomogram prediction accuracy (Figure 7).

## 4. Discussion

RCC is a common urinary tract tumour with high incidence and poor prognosis, and it is usually diagnosed at an advanced stage, leading to poor treatment effect [1–3]. At present, RCC is mainly treated by surgery, and metastatic RCC can be supplemented by drug therapy, radiotherapy, and chemotherapy. Factors affecting the prognosis of RCC include symptom, tumour type, TNM stage, Fuhrman grade, R. E. N. A. L. nephrometry score, microvascular invasion, and other factors, and TNM stage is the most valuable prognostic factor [15, 16]. This study confirmed that the symptom, tumour type, Fuhrman grade, and TNM stage are independent predictors of RCC prognosis, further confirming the previous studies.

In addition to the physiological characteristics of the tumour itself, the progress of the tumour is also closely related to the body's inflammatory response. Inflammation can lead to changes in the tumour microenvironment and promote tumour development and evolution [17]. The tumour microenvironment is a key factor affecting tumour metastasis, and immune inflammatory cells play an important role in this process [18]. RDW is closely related to various inflammatory cytokines, including interleukin-6 (IL-6), sTNF-RI, CRP, and ESP. Increased RDW levels may indicate an increase in systemic inflammation [7, 19, 20]. A meta-analysis involving 4267 patients suggested that the higher the RDW, the worse the OS, PFS, DFS, and CSS of tumour patients [21]. Lymphocytes are an important part of the immune response. They can recognise tumour cells and release cytokines to activate the immune response and kill the tumour cells. A decrease in lymphocyte count may indicate that the body's immune response to the tumour is weakened, providing conditions for the tumour to proliferate and metastasise and causing the tumour to further develop [8, 22]. Ownby et al. found that breast cancer patients with low lymphocyte levels have a high risk of recurrence [23]. Multiple lymphocyte-based inflammatory markers, such as PLR, NLR, and LMR, are independent factors for the prognosis of multiple solid tumours [24, 25]. The present study found that patients with RCC had higher RDW levels than healthy people, and lymphocyte levels were the opposite. We also confirmed that high RLR is an independent risk factor for the prognosis of patients with RCC. The level of RLR combines the overall state of the body's immunity and inflammation. RCC patients with higher RDW and fewer lymphocytes have worse postoperative prognosis. The combination of RDW and lymphocytes predicts the prognosis of cancer patients for the first time.

Fibrinogen combined with the vascular endothelial growth factor (VEGF) and platelet-derived growth factor can promote the invasion and metastasis of tumour cells through epithelial-mesenchymal transformation and induce the formation of tumour blood vessels, thereby participating in tumour progression [9, 10]. In addition, the inflammatory response can cause the body to release a variety of inflammatory factors, many of which are closely related to VEGF, which may aggravate the inflammatory response and promote the synthesis of fibrinogen in the liver [10, 11, 26]. Studies have shown that the lack of fibrinogen in mice can reduce the proliferation of tumour cells, promote the tumour necrosis, and decrease the tumour blood vessel density [27]. Chronic inflammatory response is associated with progressive malnutrition in cancer patients, and the Glasgow prognostic score composed of albumin and CRP based on inflammatory response is crucial for the prognosis of various tumours [28]. In many cancer patients, as CRP increases, albumin continues to decline and albumin reflects systemic inflammation and nutritional status [28]. Hypoalbuminemia provides a growth condition for cancer. At the same time, cancer can increase the expression of proinflammatory IL-6 and tumour necrosis factor-*α* and consequently decrease serum albumin, resulting in a vicious cycle [29]. Many studies showed that low AFR or high FAR may promote tumor infiltration, lymph node metastasis, and distant metastasis. AFR or FAR is associated with the prognosis of various cancers [9, 30]. Low AFR or high FAR often indicates a poor prognosis [9]. A meta-analysis involving 7282 patients suggests that low AFR or high FAR is associated with increased risk of death and relapse in cancer patients [30]. As a new biomarker, AFR can enhance the nutrition and inflammation sensitivity of patients, and its prognosis in patients with RCC has not been reported. Our study found that the fibrinogen levels in the patients with RCC were higher than those in the healthy people, whereas the albumin levels were lower in the patients with RCC than in the healthy people. It also confirmed the evaluation value of AFR in the patients with RCC. Low AFR indicates a poor prognosis for patients with cancer. RLR and AFR are routine laboratory results of admitted patients. These two potential biomarkers combine the body's state of inflammation, immunity, nutrition, and blood coagulation and comprehensively reflect the body's comprehensive physiological functions.

Nomogram is a visualisation of a multivariate prediction model. The model can verify its accuracy through a *C* index, ranging from 0.5 (chance) to 1 (perfect). Georg C Hutterer et al. found in the external validation cohort, and the accuracy of the nomogram constructed by symptoms and tumor size to predict distant metastasis of RCC was 85.2%, indicating that nomogram could be used as a prognostic tool for RCC patients [31]. Traditional TNM stage and Fuhrman grade are the most valuable independent prognostic factors for limited RCC. An integrated system that combines multiple independent prognostic variables can improve the accuracy of prediction [32]. The prognostic value of RLR and AFR differs in RCC patients with different tumour types and TNM stages. Thus, the appropriate patients with RCC can be stratified, and the survival time of these patients can be predicted. At the same time, we combined RLR and AFR with conventional prognostic factors to construct a nomogram. The nomogram predicts that the calibration curves for 3-year and 5-year survival rates perform well internally. Compared with other models, the *C* index for predicting OS and DFS has more accuracy in prediction, indicating that it has good predictive power, which provides a certain reference clinical value. However, the sample size of this study is small and it is a single-centre retrospective study, which may have an offset. Moreover, the nomogram results lack external verification, and the test efficiency is not high. If conditions permit, multicentre and large sample size should be combined to improve the reliability of results.

In summary, peripheral blood RLR and AFR are independent risk factors for poor prognosis in patients with RCC, and the models constructed by RLR, AFR, symptom, tumour type, TNM stage, and Fuhrman grade predict the recurrence and survival of patients with RCC.

## Figures and Tables

**Table 1:** Patient general clinical characteristics.

Characteristics	*n* (%)
*Gender*	
Male	198 (72.5)
Female	75 (27.5)

*Age* (years)	
≥65	54 (19.8)
<65	219 (80.2)

*BMI* (kg/m^2^)	
≥24	116 (42.5)
<24	157 (57.5)

*Tumour size* (D/cm)	
<7	192 (70.3)
≥7	81 (29.7)

*Tumour necrosis*	
Absent	148 (54.2)
Present	125 (45.8)

*Tumour type*	
ccRCC	203 (74.4)
chRCC	15 (5.5)
pRCC	16 (5.9)
Others	39 (14.3)

*T stage*	
*T*1	184 (67.4)
*T*2	52 (19.0)
*T*3	26 (10.6)
*T*4	8 (2.9)

*N stage*	
*N*0	258 (94.5)
*N*1	15 (5.5)

*M stage*	
*M*0	255 (93.4)
*M*1	18 (6.6)

*TNM stage*	
I	180 (65.9)
II	48 (17.6)
III	28 (10.3)
IV	17 (6.2)

*Fuhrman grade*	
*G*1	47 (17.2)
*G*2	117 (42.9)
*G*3	94 (34.4)
*G*4	15 (5.5)

**Table 2:** Associations of RLR and AFR with clinicopathological characteristics.

Characteristic	RLR			AFR		
	<8.6 (*n* = 185)	≥8.6 (*n* = 88)	*P* value	<9 (*n* = 83)	≥9 (*n* = 190)	*P* value
Age, (years)	50.83 ± 13.08	56.09 ± 12.25	0.002	56.19 ± 12.07	50.92 ± 13.14	0.020

Gender			0.597			0.009
** **Male	136	62		129	69	
** **Female	49	26		61	14	

BMI, (kg/m^2^)	24.32 ± 4.63	22.07 ± 3.25	<0.001	23.34 ± 4.18	23.71 ± 4.44	0.527

HGB, (g/L)	133.98 ± 17.04	119.87 ± 20.89	<0.001	121.53 ± 23.25	132.88 ± 16.51	<0.001

Ca, (mmol/L)	2.30 ± 0.10	2.29 ± 0.22	0.640	2.30 ± 0.20	2.30 ± 0.13	0.776

Creatinine, (umoI/L)	84.50 ± 26.21	84.64 ± 25.29	0.968	92.57 ± 32.11	81.04 ± 21.80	0.003

Ccr, (ml/min)	89.97 ± 24.58	81.02 ± 17.55	0.001	77.73 ± 20.50	91.17 ± 22.76	<0.001

Basic disease			0.008			0.300
** **No	144	55		57	142	
** **Yes	41	33		26	48	

Symptom			0.013			0.001
** **No	99	33		28	104	
** **Yes	86	55		55	86	

Surgery			0.156			0.006
** **Endoscopic	143	61		53	154	
** **Open	42	27		30	39	

Site			0.737			0.597
** **Left	99	49		47	101	
** **Right	86	39		36	89	

Tumour size (D/cm)	4.50 (3.45 – 3.05)	5.90 (3.50 – 9.00)	0.010	7.00 (4.00 – 9.50)	4.10 (3.00 – 6.00)	<0.001

Tumour necrosis			0.482			0.186
** **Absent	103	45		50	98	
** **Present	82	43		33	92	

Tumour type			0.044			0.034
** **ccRCC	145	59		55	149	
** **nccRCC	40	29		28	41	

TNM stage			0.027			<0.001
** **I	130	50		33	147	
** **II	33	15		19	29	
** **III	13	15		19	9	
** **IV	9	8		12	5	

Fuhrman grade			0.121			0.001
** ** *G*1 + *G*2	117	47		38	126	
** ** *G*3 + *G*4	68	41		45	64	

**Table 3:** Univariable analysis of clinicopathologic variables in relation to OS and DFS in RCC patients.

Parameter	OS		DFS	
	HR (95% CI)	*P*	HR (95% CI)	*P*
Age (years)		0.026		0.050
** **<65	1 (referent)		1 (referent)	
** **≥65	1.984 (1.085 – 3.627)		1.783 (1.000 – 3.181)	

Gender		0.100		0.341
** **Male	1 (referent)		1 (referent)	
** **Female	0.546 (0.266 – 1.123)		0.740 (0.399 – 1.374)	

BMI (kg/m^2^)		0.226		0.829
** **≥24	1 (referent)		1 (referent)	
** **<24	1.426 (0.803 – 2.534)		1.060 (0.626 – 1.793)	

HGB (g/L)		0.001		0.004
** **≥120	1 (referent)		1 (referent)	
** **<120	2.588 (0.224 – 0.681)		2.201 (1.296 – 3.740)	

Ca (mmol/L)		0.019		0.134
** **<2.4	1 (referent)		1 (referent)	
** **≥2.4	2.134 (1.468 – 4.457)		1.630 (0.860 – 3.087)	

Creatinine (*μ*moI/L)		0.060		0.075
** **<90	1 (referent)		1 (referent)	
** **≥90	1.700 (0.979 – 2.952)		1.609 (0.953 – 2.716)	

Ccr (ml/min)		<0.001		0.001
** **>80	1 (referent)		1 (referent)	
** **≤80	2.968 (1.670 – 5.274)		2.466 (1.452 – 4.188)	

Basic disease		0.425		0.381
** **No	1 (referent)		1 (referent)	
** **Yes	1.277 (0.707 – 2.307)		1.284 (0.734 – 2.244)	

Symptom		0.001		0.001
** **No	1 (referent)		1 (referent)	
** **Yes	2.751 (1.487 – 5.090)		2.718 (1.525 – 4.845)	

Surgery		0.001		0.002
** **Endoscopic	1 (referent)		1 (referent)	
** **Open	2.613 (1.503 – 4.537)		2.269 (1.336 – 3.855)	

Site		0.640		0.524
** **Left	1 (referent)		1 (referent)	
** **Right	0.876 (0.503 – 1.525)		0.843 (0.498 – 1.426)	

Tumour size (D/cm)		<0.001		<0.001
** **<7	1 (referent)		1 (referent)	
** **≥7	5.352 (3.010 – 9.517)		4.196 (2.475 – 7.113)	

Tumour necrosis		0.779		0.156
** **Absent	1 (referent)		1 (referent)	
** **Present	0.924 (0.530 – 1.608)		0.677 (0.395 – 1.161)	

Tumour type		<0.001		<0.001
** **ccRCC	1 (referent)		1 (referent)	
** **nccRCC	3.235 (1.858 – 5.634)		3.080 (01.821 – 5.208)	

TNM stage		<0.001		<0.001
** **I	1 (referent)		1 (referent)	
** **II	4.210 (1.951 – 9.086)		3.375 (1.678 – 6.790)	
** **III	9.848( 4.612 – 21.026)		8.305 (4.164 – 16.562)	
** **IV	13.634 (6.088 – 30.531)		10.427 (4.787 – 22.713)	

Fuhrman grade		<0.001		<0.001
** ** *G*1 + *G*2	1 (referent)		1 (referent)	
** ** *G*3 + *G*4	4.548 (2.455 – 8.424)		3.250 (1.885 – 5.603)	

RLR		<0.001		<0.001
** **<8.6	1 (referent)		1 (referent)	
** **≥8.6	3.350 (1.923 – 5.834)		3.012 (1.783 – 5.077)	

AFR		<0.001		<0.001
** **≥9	1 (referent)		1 (referent)	
** **<9	6.816 (3.729 – 12.458)		5.957 (3.428 – 10.350)	

**Table 4:** Multivariable analysis of clinicopathologic variables in relation to OS and DFS in RCC patients.

Parameter	OS		DFS	
	HR (95% CI)	*P*	HR (95% CI)	*P*
Symptom		0.036		0.020
** **No	1 (referent)		1 (referent)	
** **Yes	2.027 (1.046 – 3.927)		2.051 (1.117 – 3.764)	

Tumour type		<0.001		<0.001
** **ccRCC	1 (referent)		1 (referent)	
** **nccRCC	4.445 (2.387 – 8.279)		2.834 (1.652 – 4.859)	

TNM stage		<0.001		<0.001
** **I	1 (referent)		1 (referent)	
** **II	2.816 (1.268 – 6.256)		2.118 (1.032 – 4.349)	
** **III	5.994 (2.604 – 13.800)		4.161 (1.954 – 8.861)	
** **IV	9.980 (4.039 – 24.657)		6.253 (2.649 – 14.759)	

Fuhrman grade		<0.001		0.003
** ** *G*1 + *G*2	1 (referent)		1 (referent)	
** ** *G*3 + *G*4	3.409 (1.817 – 6.705)		2.326 (1.323 – 4.090)	

RLR		0.007		0.013
** **<8.6	1 (referent)		1 (referent)	
** **≥8.6	2.255 (1.242 – 4.093)		1.969 (1.152 – 3.367)	

AFR		0.019		0.008
** **≥9	1 (referent)		1 (referent)	
** **<9	2.349 (1.153 – 4.787)		2.357 (1.254 – 4.433)	

## Data Availability

All primary data are available from the corresponding author upon reasonable request.
